# Antigravitropic PIN polarization maintains non-vertical growth in lateral roots

**DOI:** 10.1038/s41477-023-01478-x

**Published:** 2023-09-04

**Authors:** Suruchi Roychoudhry, Katelyn Sageman-Furnas, Chris Wolverton, Peter Grones, Shutang Tan, Gergely Molnár, Martina De Angelis, Heather L. Goodman, Nicola Capstaff, James P. B. Lloyd, Jack Mullen, Roger Hangarter, Jiří Friml, Stefan Kepinski

**Affiliations:** 1 School of Biology, University of Leeds, Leeds, UK; 2 Ohio Wesleyan University, Delaware, OH USA; 3 Institute of Science and Technology, Vienna, Austria; 4 University of Western Australia, Perth, Western Australia Australia; 5 Department of Bioagricultural Sciences & Pest Management, Colorado State University, Fort Collins, CO USA; 6 Department of Biology, Indiana University, Bloomington, IN USA; 7 Present Address: Department of Biology, Duke University, Durham, NC USA; 8 Present Address: Umeå Plant Science Centre, Umeå, Sweden; 9 Present Address: Department of Applied Genetics and Cell Biology, University of Natural Resources and Life Sciences (BOKU), Vienna, Austria; 10 Present Address: Tropic Biosciences Ltd, Norwich Research Park Innovation Centre, Norwich, UK; 11 Present Address: Department of Science, Innovation and Technology, UK Government, London, UK

**Keywords:** Auxin, Plant morphogenesis

## Abstract

Lateral roots are typically maintained at non-vertical angles with respect to gravity. These gravitropic setpoint angles are intriguing because their maintenance requires that roots are able to effect growth response both with and against the gravity vector, a phenomenon previously attributed to gravitropism acting against an antigravitropic offset mechanism. Here we show how the components mediating gravitropism in the vertical primary root—PINs and phosphatases acting upon them—are reconfigured in their regulation such that lateral root growth at a range of angles can be maintained. We show that the ability of *Arabidopsis* lateral roots to bend both downward and upward requires the generation of auxin asymmetries and is driven by angle-dependent variation in downward gravitropic auxin flux acting against angle-independent upward, antigravitropic flux. Further, we demonstrate a symmetry in auxin distribution in lateral roots at gravitropic setpoint angle that can be traced back to a net, balanced polarization of PIN3 and PIN7 auxin transporters in the columella. These auxin fluxes are shifted by altering PIN protein phosphoregulation in the columella, either by introducing PIN3 phosphovariant versions or via manipulation of levels of the phosphatase subunit PP2A/RCN1. Finally, we show that auxin, in addition to driving lateral root directional growth, acts within the lateral root columella to induce more vertical growth by increasing RCN1 levels, causing a downward shift in PIN3 localization, thereby diminishing the magnitude of the upward, antigravitropic auxin flux.

## Abstract (web summary)

Lateral roots grow at specific angles with respect to gravity. Two auxin transporters, PIN3 and PIN7, in gravi-sensing cells establish hormone gradients that balance downward and upward growth to generate stable, non-vertical roots.

## Main

Gravity is one of the most fundamental environmental signals controlling plant development and certainly the most constant. The capacity for gravity-directed growth, known as gravitropism, ensures that shoots typically grow upwards and roots grow downwards, allowing light interception and gas exchange above ground, and water and nutrient uptake below. These processes of resource capture are enhanced enormously by the production of lateral root and shoot branches that grow out from the main root–shoot axis at non-vertical angles. Importantly, these branches are often maintained at specific angles with respect to gravity, independently of the main or parent axis from which they originate. These patterns of growth in primary and lateral organs are most easily understood in the context of the gravitropic setpoint angle (GSA) concept^1^. The GSA is the angle at which an organ is maintained with respect to gravity by the action of gravitropism. Vertically growing organs have a GSA of 0° if they are growing towards the centre of the Earth and 180° if growing away, with non-vertical branches having GSAs between these two extremes.

To alter growth according to gravity, plant organs must have the capacity to perceive their orientation within the gravity field and to regulate elongation on their upper and lower sides differentially. These processes are well described by the starch–statolith model of graviperception and the Cholodny–Went model of tropic growth. In the first, the sedimentation of starch-rich amyloplasts within specialized statocyte cells provides information on the organ’s angle with respect to gravity^2–4^. This information is translated into tropic growth through the asymmetric redistribution of the hormone auxin to the lower side of the organ^5–9^. Here, according to the Cholodny–Went model, auxin inhibits cell elongation in the root and promotes cell elongation in the shoot, driving downward and upward growth, respectively^5,6^.

The starch–statolith and Cholodny–Went models are linked by the action of the PIN family of auxin efflux carrier proteins and in particular, PIN3 and PIN7 (refs. ^7,10,11^). In *Arabidopsis*, both PIN3 and PIN7 are expressed in the columella statocyte cells where their subcellular distribution is dependent upon the orientation of the root tip. In a primary root growing vertically, PIN3 and PIN7 localization is essentially apolar, but upon gravistimulation, both PIN3 and PIN7 can become rapidly relocalized to accumulate on the lateral, lower-most face of the columella cells, increasing the downward flux of auxin^7,10,11^. From the root cap, auxin is transported shootward by PIN2 via the epidermis to the elongation zone (EZ) where cell expansion is regulated^12–14^. Thus together, the starch–statolith and Cholodny–Went models, with the more recent addition of PIN-based auxin transport, are sufficient to account for the maintenance of the vertical growth typically observed in the primary root and shoot.

Non-vertical GSAs present an intriguing problem because, to maintain a non-vertical growth angle, root and shoot branches must by definition have the capacity to reorient their growth both with and against gravity^15–17^. It has previously been shown that non-vertical GSAs are the result of an auxin-dependent antigravitropic offset (AGO or, simply, offset) mechanism that counteracts the underlying gravitropic response in root and shoot branches such that stable, angled growth occurs when both growth components are balanced^16^. Using auxin treatment and mutants affected in either auxin homeostasis or response, it was also shown that auxin induced more vertical GSAs by diminishing the relative magnitude of the AGO^16^.

The fact that AGO activity is auxin dependent hints at a mechanism involving Cholodny–Went-type tropic response. Consistent with this idea, mutation of the columella-expressed auxin efflux carriers PIN3, PIN4 and PIN7 has been shown to affect lateral root GSA^18,19^. Further, these PIN proteins also show specific expression patterns throughout the development of the lateral root^18–20^. In *Arabidopsis*, lateral roots emerge at a near horizontal orientation (stage I/type 1) and following the development of differentiated columella and a distinct EZ that marks the acquisition of gravicompetence, undergo a brief period of downward growth (stage II/type 2) (refs. ^15,18,19,21^). From this point, lateral roots gain the capacity to stably maintain non-vertical GSAs and gradually transition through progressively more vertical GSA states (stages III–V/types 3–6). Stage III, sometimes referred to as the plateau phase, is particularly important in determining the extent of radial expansion of the root system^15,18^. PIN3:green fluorescent protein (GFP) expression in the columella is apparent from stage I onwards but begins to decline after stage III. In contrast, PIN4:GFP and PIN7:GFP are undetectable in emerging lateral roots (stages I and II), with PIN7 expression in the columella becoming apparent in stage III and PIN4 detected in the columella from approximately stage IV onwards^18,19,22^. On the basis of these distinctive expression patterns it was suggested that non-vertical GSAs might simply be the result of reduced gravitropic competence, arising from the fact that PIN protein expression levels in the columella of the lateral root are lower than those in the primary root^18,19^. While these spatiotemporal PIN expression patterns are likely to be highly relevant to lateral root GSA regulation, a model based solely on a lack of gravitropic competence is incompatible with the data supporting the GSA concept, most strikingly, the capacity of lateral roots to grow upwards to regain their GSA.

In this Article, we have used molecular and genetic tools to reveal the mechanisms controlling GSA in the *Arabidopsis* lateral root. We show that GSA maintenance is underpinned by the control of both upward antigravitropic and downward gravitropic auxin fluxes in a manner consistent with the Cholodny–Went model. Specifically, we show that the ability of lateral roots to bend both downward and upward to maintain GSA is driven by control of cell elongation on the lower side of the reorientating root. This organ-level behaviour is consistent with the response to the observed angle-dependent variation in downward gravitropic auxin transport, where the magnitude of flux decreases closer to the vertical, acting against the response to an upward, antigravitropic auxin flux that is more or less constant for a given GSA. These patterns of auxin distribution in the root tip are dependent on the subcellular localization of PIN3 and PIN7, which not only mediate gravitropic response, but also constitute the antigravitropic offset. In this context, growth at GSA is characterized by a net balanced capacity for upward and downward PIN-mediated auxin transport from the lateral root columella. Finally, we show that the protein phosphatase 2A subunit ROOTS CURL IN NPA1 (RCN1) acts upstream of PIN3 to promote an upper to lower side shift in the polarity of PIN3, but not PIN7, and further that auxin induces more vertical GSA in lateral roots by increasing RCN1 levels in the columella, thereby diminishing the magnitude of the upward, AGO auxin flux.

## Results

### Gravitropic auxin transport in the *Arabidopsis* lateral root is offset by an antagonistic auxin flux

**Fig. 1:**
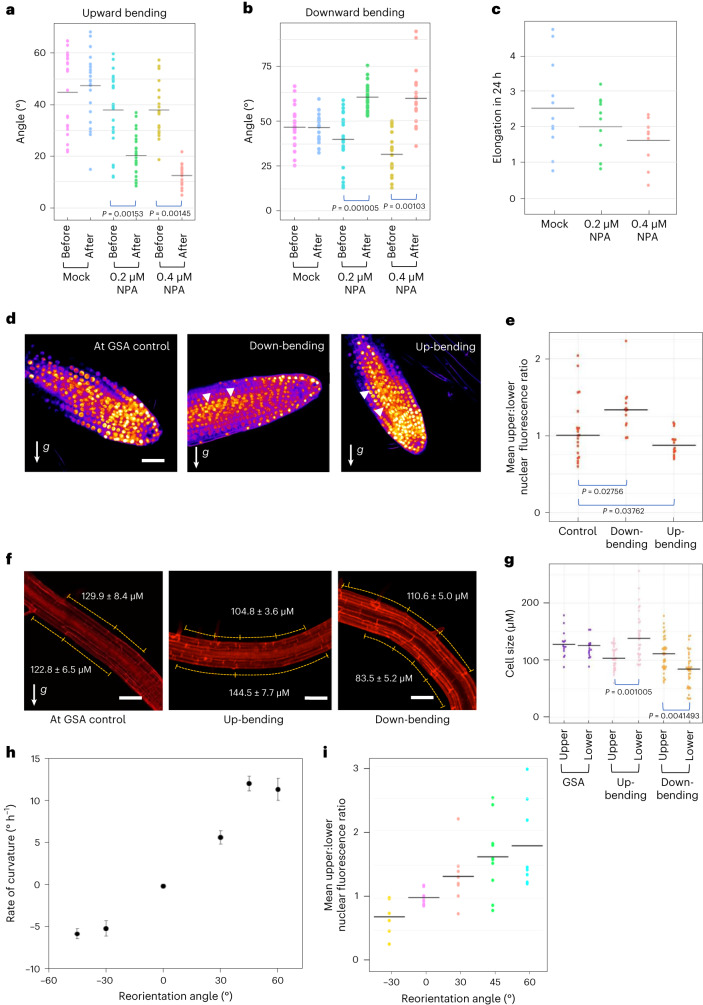
Lateral root graviresponse is angle dependent and driven by auxin transport-dependent auxin asymmetry. **a**,**b**, Mean GSA of mock- and NPA-treated stage III lateral roots growing at GSA and 24 h after reorientation by 30°. Treatment with 0.2 µM and 0.4 µM NPA inhibits lateral root reorientation in both upward (**a**) and downward (**b**) directions. *n* = 25–37 roots for each treatment from 3 biologically independent experiments. One-way ANOVA followed by post hoc Tukey’s HSD test revealed *F*(5) = 13.7890 for **a** and 25.8968 for **b** gave *P* = 1.102 × 10^−16^ for **a** and **b**. **c**, Change in length of stage III lateral roots during mock and NPA treatments. The growth rates of NPA-treated roots are not significantly different from mock-treated roots (*P* = 0.0838) *n* = 10 roots per treatment from 3 biologically independent experiments. **d**, Visualization of auxin fluxes using the auxin reporter DII-Venus in upward and downward reoriented lateral roots. Scale bar, 20 μm. **e**, Ratio of mean nuclear fluorescence between upper and lower epidermal cells of stage III lateral roots gravistimulated 30° above or below their GSA using the auxin reporter DII-Venus. Note: to aid understanding, the colloquial terms up- and down-bending are used as short descriptors of lateral roots undergoing negative (upward) and positive (downward) gravitropic response respectively. White arrowheads indicate increased nuclear fluorescent signal on the upper and lower sides of downward and upward bending lateral roots respectively. *n* = 10–15 roots analysed for each angle. Roots were reoriented from their GSA on the rotating stage of a vertical confocal microscope and imaged 60 min post reorientation. One-way ANOVA followed by post hoc Tukey’s HSD test revealed *F*(2) = 6.325 gave *P* value of 0.0039. **f**,**g**, Quantification of atrichoblast epidermal cell lengths at upper and lower sides of reorientated lateral roots. In upward-bending lateral roots, epidermal cells on the bottom half of the root are significantly longer than those on the upper side (**f** middle panel and **g**). In contrast, in downward-bending lateral roots, epidermal cells on the bottom of the root are significantly shorter in length than those on the upper side (**f** third panel and **g**). Scale bars, 50 μm. *n* = 20–35 for each group from 3 biologically independent experiments. One-way ANOVA followed by post hoc Tukey’s HSD test revealed *F*(5) = 13.7890 gave *P* value of 5.2996 × 10^−11^. **h**, Kinetics of gravitropism in lateral roots growing at oblique orientations. Curvature was measured in terms of stage rotation for roots maintained 30° from their original orientation by a feedback system following either an upward reorientation, resulting in positive gravitropism, or a downward reorientation, resulting in negative gravitropism (mean ± standard error of the mean, *n* = 18–22). **i**, Ratio of mean nuclear DII-Venus fluorescence between upper and lower epidermal cells of stage III lateral roots gravistimulated above or below their GSA by different angles. Roots were gravistimulated for 90 min before imaging. *n* = 8–10 roots for each angle of stimulation from 3 biologically independent experiments. Source data

The control of auxin distribution across the root tip is central to gravitropism and the maintenance of a typically vertical GSA in the primary root. To explore the role of auxin transport in the maintenance of non-vertical GSAs, we tested the effect of the auxin transport inhibitor NPA^23^ on both upward and downward gravitropic growth in reorientated lateral roots. In these experiments, lateral roots treated with either 0.2 μM or 0.4 μM NPA failed to return to their original GSA after 24 h following rotation by 30° either above or below their GSA, albeit adopting a more vertical GSA. (Fig. 1a,b). At both concentrations, the growth rates of lateral roots, although reduced, are not statistically significantly different from the wild type (WT) in our growth conditions (Fig. 1c)^16^. These data therefore indicate that auxin transport is necessary for both upward and downward gravity induced growth curvatures.

**Extended Data Fig. 1:**
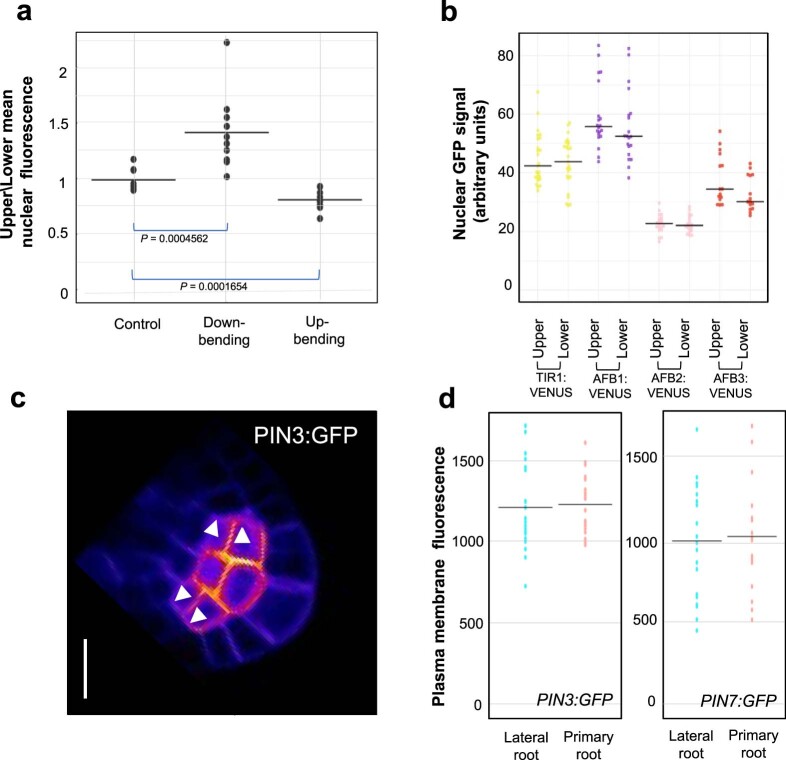
Analysis of auxin signalling and PIN polarity in lateral root columella cells. (**a**) Ratio of nuclear R2DII signal across upper and lower epidermal cells in lateral roots at GSA (control) and reorientated upwards and downwards. Images were taken 90 mins post reorientation Bars represent standard error of mean nuclear fluorescence. n = 12–15 roots for each orientation from 3 biologically independent experiments. One way ANOVA revealed F stat (2) = 22.25654 with a p value = 0.001346. (**b**) Statistical analysis using pairwise two-tailed *T*-tests revealed no significant difference in mean nuclear fluorescence of TIR1/AFB:Venus in atrichoblast cells on the upper and lower side of stage III lateral roots. n = 39–51 nuclei analysed for each transgenic line across 3 biologically independent experiments. **(c**) Outer membranes (white arrowheads) of upper and lower cells of the central columella used for quantification of PIN polarity in a single stack of a PIN3:GFP lateral root. Scale bar = 5 μm. (**d**) Quantification of PIN3/7::GFP fluorescence levels in plasma membranes of columella cells from stage III lateral and primary roots. n = 21–25 roots for each transgenic line quantified from 3 biologically independent experiments. Pairwise two tailed T-tests revealed no significant differences in membrane fluorescence levels.

To analyse auxin distribution and response in lateral roots growing at their GSA, and following gravistimulation above and below their GSA, we used the reporters DII-Venus and the ratiometric DII-Venus variant, R2D2 (refs. ^24,25^). In lateral roots growing at their GSA, these reporters indicated no significant difference in auxin levels between the upper and lower halves of lateral roots (Fig. 1d,e and Extended Data Fig. 1a). The inference of auxin levels from DII-Venus and R2D2 in this context is further supported by the lack of variation in TIR1/AFB auxin receptor levels across the lateral root, quantified by TIR1:Venus, AFB1:Venus, AFB2:Venus and AFB3:Venus translational reporter expression (Extended Data Fig. 1b)^26^. In lateral roots reorientated above their GSA (and bending downwards), quantification of DII-Venus (Fig. 1d,e) and R2D2 (Extended Data Fig. 1a) signals 90 min post reorientation indicated higher levels of auxin accumulation on the lower side of lateral roots (Fig. 1d,e and Extended Data Fig. 1a). Conversely, in roots displaced below their GSA (and thus bending upwards), DII-Venus (Fig. 1d,e) and R2D2 (Extended Data Fig. 1a) signals indicated higher levels of auxin accumulation on the upper side of the lateral root, in the direction of tropic growth. Together, these data indicate that the maintenance of non-vertical GSAs is auxin transport dependent and entirely compatible with the Cholodny–Went model of tropic growth.

To understand how these patterns of auxin distribution in reorientated lateral roots relate to the asymmetric cell elongation driving tropic curvature, we measured atrichoblast epidermal cell lengths across the upper and lower sides of stage III lateral roots both at GSA and following reorientation. In keeping with the lack of auxin asymmetry in lateral roots growing at GSA, we found that there were no significant differences in cell lengths between the upper and lower sides of the root (Fig. 1f,g). In downward-bending lateral roots, epidermal cells on the lower side of the root were significantly shorter than those at the upper side, consistent with the asymmetric auxin accumulation in these cells and resulting auxin-mediated growth inhibition^27^ in the lower half of the root (Fig. 1f,g). In upward-bending roots, we observed that the cells on the lower side of the root were significantly longer than those on the upper side, and, interestingly, they were also longer than epidermal cells in roots growing at their GSA (Fig. 1f,g). Importantly, epidermal cells on the upper side of lateral roots undergoing either upward or downward tropic growth responses did not differ significantly in length. These data indicate that tropic growth in lateral roots is driven principally by control of cell elongation on the lower side of the root. This suggests a mechanism in which the maintenance of non-vertical GSAs depends upon stimulation angle-dependent variation in the gravitropic response on the lower side of the lateral root against a more constant and angle-independent antigravitropic component on the upper side.

To test if lateral root gravitropic responses are angle dependent, we used a feedback-regulated system^28^ to constrain stage III lateral roots at 30° and 45° below, and 30°, 45°, 60° and 90° above their GSA. Constraint at 30° above or below GSA (mean GSA 63°, standard deviation 7°) elicited almost identical rates of downward and upward bending respectively (Fig. 1h) Increasing the angle of reorientation to 45° above GSA led to a more than doubling of the rate of curvature (Fig. 1h), confirming that, similar to primary roots^28^, lateral roots are able to respond to gravity in an angle-dependent manner. Using the DII-Venus reporter, we found that these differences in angle-dependent reorientation kinetics were reflected in the magnitude of asymmetric auxin gradients across the upper and lower halves of lateral roots reoriented at different angles (Fig. 1i), providing a mechanistic explanation for the angle-dependent graviresponse.

### Non-vertical GSAs arise from a net symmetry in the polarity of PIN3 and PIN7 in the lateral root columella

**Fig. 2:**
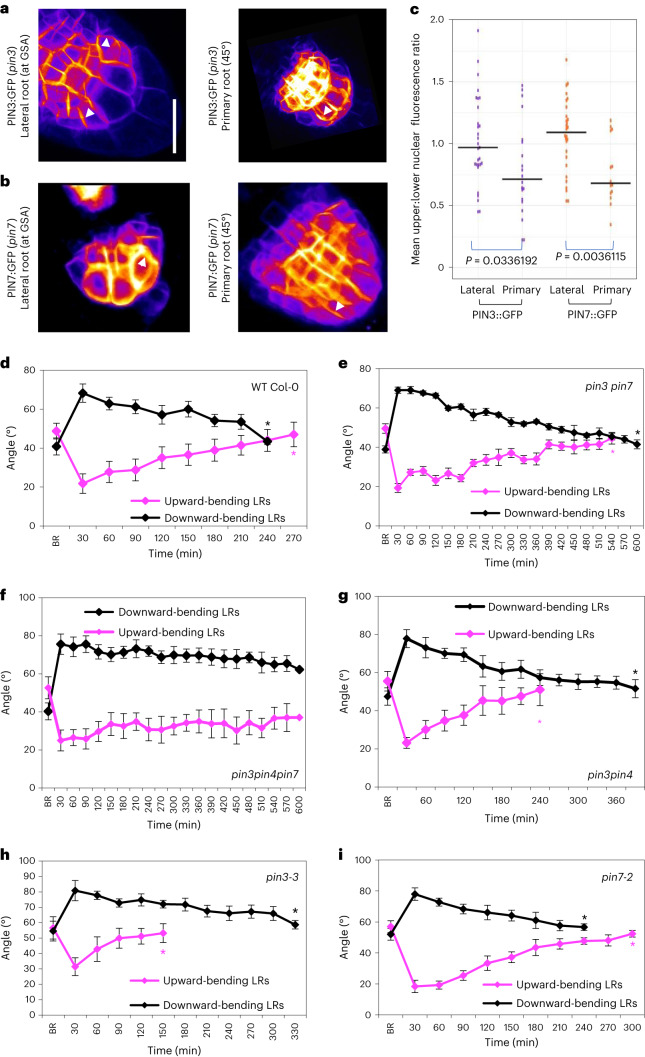
PIN polarity distribution in lateral roots. **a**,**b**, Comparison of PIN polarity in lateral roots at their GSA and primary roots reoriented by 45° roots in seedlings expressing PIN3:GFP (**a**) and PIN7:GFP (**b**). Fluorescence was measured on upper and lower membranes of outer columella cells as indicated in Extended Data Fig. 1c. PIN3 shows a slight polarity towards the lower cell membrane (**a**), while, in contrast, PIN7:GFP shows enhanced polarity towards the upper membrane (**b**) in lateral roots growing at their GSA. **c**, However, both PIN3:GFP (**a**) and PIN7:GFP (**b**) are predominantly polarized towards the lower plasma membrane in primary roots reoriented by ~45° for 30 min. Scale bar, 15 μm (**a** and **b**). *n* = 20–25 roots from 3 biologically independent experiments. Statistical analysis was carried out using a pairwise two-tailed *t*-test. **d**–**i**, Comparison of reorientation kinetics in lateral roots of 12-day-old WT Col-0 (**d**), *pin3 pin7* (**e**), *pin3pin4pin7* (**f**), *pin3pin4* (**g**), *pin3-3* (**h**) and *pin7-2* (**i**) seedlings gravistimulated both above and below their GSA. BR represents GSA before reorientation. Average GSA of 10–12 upward- and downward -bending stage III lateral roots was quantified after reorientation until the roots were within 5° of their original GSA. Black asterisks indicate the timepoint at which angles were recovered for downward-bending roots, while magenta asterisks indicate the timepoint at which angles were recovered for upward-bending roots. *pin3* and *pin3pin4* lateral roots reorientate upwards significantly faster (**g** and **h**), while *pin7* lateral roots reorientate downwards at a faster rate (**i**). WT Col-0 control lateral roots reorientate back to their GSA in both directions in approximately 6 h (**d**). In contrast, reorientation in both directions is delayed in the *pin3 pin7* double mutant (**e**) and is virtually negligible in the *pin3pin4pin7* triple mutant (**f**). *n* = 15–21 roots at all timepoints (**d**–**i**) from 3 biologically independent experiments. Bars in **d**–**i** represent standard error of the means.

In *Arabidopsis*, lateral roots emerge at a near horizontal orientation (stage I/type 1) and following the development of differentiated columella and a distinct EZ that marks the acquisition of gravicompetence, undergo a brief period of downward growth (stage II/type 2) (refs. ^15,18,19,21^). From this point, lateral roots gain the capacity to stably maintain non-vertical GSAs and gradually transition through progressively more vertical GSA states (stages III–V/types 3–6). Of these phases of gravity-dependent growth, stage III, sometimes referred to as the plateau phase, is particularly important in determining the extent of radial expansion of the root system^15,18^. Both PIN3 and PIN7 have been previously described to play a major role in translating information on the direction of gravity into asymmetric auxin fluxes by their gravity-induced polarization^7,10,11^ and are expressed in stage III lateral roots, from which point GSAs are robustly maintained^18,20^. We therefore studied the localization and distribution of these PINs in *Arabidopsis* lateral roots growing at non-vertical GSAs using vertical-stage confocal microscopy^29^. In these experiments we measured the ratio of GFP signal in the plasma membranes on the upper and lower sides of outermost flanking cells (Extended Data Fig. 1c). We found that PIN3, expressed mainly in the top two tiers of columella cells, was targeted to both upper and lower plasma membranes, but was targeted to the lower membrane in a slightly greater proportion of columella cells (Fig. 2a,c). PIN7 exhibited a distinctly different pattern, being targeted to the upper plasma membrane in over 50% of lateral roots analysed (Fig. 2b,c). In contrast with lateral roots at GSA, in primary roots placed non-vertically (~45°), both PIN3 and PIN7 polarized predominantly towards the lower side of the columella (Fig. 2a–c) 30 min after reorientation, consistent with previous studies^10^. We also quantified the mean plasma membrane fluorescence levels of PIN3:GFP and PIN7:GFP in columella cells of stage III lateral and primary roots imaged under the same settings. These experiments verified that there were no differences in PIN3 and PIN7 protein levels within lateral and primary columella cell membranes (Extended Data Fig. 1d).

Thus, PIN proteins in lateral roots have polarization patterns that are distinct from those in primary roots, potentially providing an explanation for the symmetry in auxin distribution in roots growing at their GSA.

**Extended Data Fig. 2:**
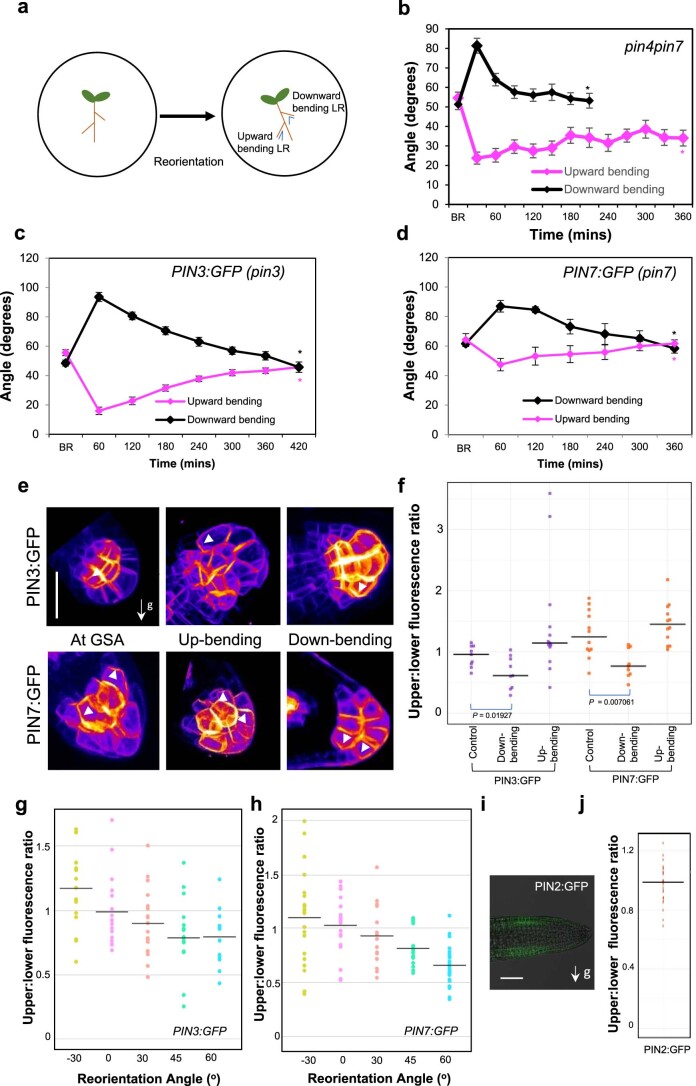
Quantification of the kinetics of gravitropic response in lateral roots of single and multiple *pin* mutants. (**a**) Schematic representation of seedling reorientation for analysis of reorientation kinetics in WT and *pin* mutants. Plates were reoriented by 30° angles and tip angles of roots placed above and below their GSA (denoted in blue) were quantified at specified time intervals. (**b**) Reorientation kinetics of 12-day-old lateral roots in the *pin4pin7* double mutant. *pin4pin*7 lateral roots bend downwards rapidly, but show delayed upward bending presumably due to the loss of PIN7. (**c, d**) Reorientation kinetics of stage III lateral roots in the PIN3:GFP (C) and PIN7:GFP (D) transgenic lines. Lateral roots return to their original GSA in approximately 6 hours after reorientation in both directions. (B-D) n = 17–21 lateral roots at each time point from 3 biologically independent experiments (**e, f**) PIN polarity changes in 12-day old lateral roots reorientated in upward and downward directions. (**e**) PIN3:GFP and PIN7:GFP are predominantly dipolar (PIN3) and apolar (PIN7) in lateral roots at their GSA (right panels). In lateral roots reorientated below their GSA, both PIN3 and PIN7 polarise towards the upper side of columella cells (middle panels) whereas in roots displaced above their GSA, both PINs polarise towards the lower side of columella cells (left panels). White arrowheads indicate polar localisation of PIN:GFP signal. Scale bar = 5 μm. n = 12–15 roots at each reorientation from 3 biologically independent experiments. One way ANOVA revealed F stat (2) = 4.5096 with a P value = 0.0201 for PIN3:GFP and F stat (2) = 10.4650 with a P value = 0.0004 for PIN7:GFP. (**g, h**) Quantification of upper/ lower mean PIN3/7:GFP signal across external columella cell membranes in lateral roots at GSA and reorientated upwards and downwards at defined angles. Negative angles denote reorientation below GSA. Bars represent standard error of the means. (**i**) PIN2:GFP signal is not differentially expressed across upper and lower sides of lateral roots (right panel). Scale bar = 20 μm. (**j**) Quantification of PIN2:GFP upper/lower epidermal signal in trichoblasts across upper and lower epidermal cell files.

To explore the significance of these cell biological observations, we examined the kinetics of graviresponse in *pin3-3* and *pin7*-2 single and double mutants (schematic of the experimental setup is shown in Extended Data Fig. 2a). Upon reorientation by 30° above and below GSA, we found that lateral roots of the *pin3 pin7* double mutant were severely delayed in returning toward their GSA compared with WT, although both upward and downward tropic growth was still apparent (Fig. 2d,e). Because previous studies have shown that PIN4 expression domains expand into the columella in a compensatory manner in the *pin3* and *pin7* mutant backgrounds^30^, we also examined the graviresponse in the *pin3 pin4 pin7* triple mutant and found the response to be virtually absent over a 10 h timeframe (Fig. 2f).

The graviresponse kinetics of the *pin3* and *pin7* single mutants were particularly interesting. We found that lateral roots of *pin3* seedlings reorientated upwards significantly faster than downwards (Fig. 2h), while the reverse was true for lateral roots of *pin7* seedlings, albeit to a lesser degree (Fig. 2i). In contrast, the graviresponse of *pin3* and *pin7* mutants complemented with PIN3:GFP (ref. ^31^) and PIN7:GFP (ref. ^32^) was similar to WT Col-0 (Extended Data Fig. 2c,d). We also tested the response of the *pin3 pin4* double mutant, which, similar to the *pin3* single mutant, exhibited a much more rapid upward relative to downward tropic growth (Fig. 2g). In contrast, the *pin4pin7* double mutant displayed rapid downward tropic growth, while upward tropic growth was delayed, presumably due to the loss of PIN7 (Extended Data Fig. 2b). These data indicate that the rapid upward bending associated with loss of PIN3 function requires PIN7 and are thus consistent with the observed subcellular polarity bias of PIN7 and, to some extent, PIN3.

To determine if the changes in auxin distribution observed in lateral roots under reorientation (Fig. 1d,e and Extended Data Fig. 1a) were reflected in shifts in PIN localization, we gravistimulated lateral roots both above and below their GSA and examined PIN3:GFP and PIN7:GFP localization in the columella by vertical-stage confocal microscopy. For lateral roots growing at their GSA, the average upper/lower ratio for PIN3:GFP was approximately equal to 1, whereas the ratio was slightly higher (1.2) for PIN7:GFP (Extended Data Fig. 2e,f) as described above (Fig. 2c). Stimulation above GSA (downward bending) shifted the polarity of both PIN3 and PIN7 to a predominantly basal localization, similar to that in primary roots (Extended Data Fig. 2e,f). In contrast, where lateral roots were reoriented below their GSA (upward bending), we observed an increased signal at the upper plasma membrane relative to the lower side for both PIN3:GFP and PIN7:GFP lateral roots (Extended Data Fig. 2e,f). Further, we observed that these shifts in PIN polarity occurred in an angle-dependent manner, particularly in roots reoriented above their GSA and bending downwards (Extended Data Fig. 2g,h). Thus, these changes in PIN3 and PIN7 polarity are consistent with the observed R2D2 and DII-Venus data (Fig. 1d,e and Extended Data Fig. 1a) demonstrating auxin redistribution during lateral root gravistimulation.

In addition to PIN3 and PIN7 activity in the columella, root graviresponse also requires the action of PIN2 in the epidermis to drive the basipetal flow of auxin away from the root tip^33^. We therefore also studied PIN2:GFP expression in lateral roots growing at their GSA to check for any differential expression of PIN2 that might contribute to the regulation of growth angle. This analysis did not reveal differences in PIN2 expression between upper and lower halves of lateral roots (Extended Data Fig. 2i,j).

### PIN protein retention at the plasma membrane differs between upper and lower faces of repolarizing lateral root statocytes

**Fig. 3:**
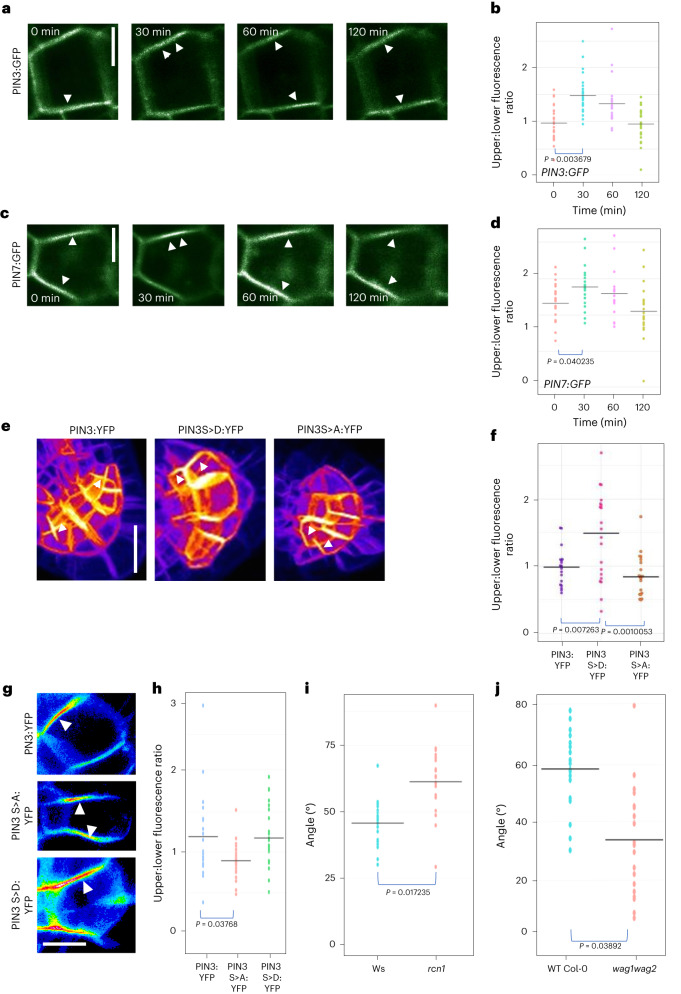
PIN phosphorylation affects PIN polarity and redistribution kinetics in lateral root statocytes. **a**–**d**, PIN polarity distribution in columella cells of lateral roots rotated around their axis of growth by 90° (‘flip assays’; for a diagrammatic description of the experiment, see Extended Data Fig. 3a). In all panels, the former upper side of the columella cell is towards the top of the page. Post flip, phosphorylated PIN3 and PIN7 are retained on the upper plasma membrane for approximately 30 min longer than lower side unphosphorylated PINs. PIN3 (**a** and **b**) and PIN7 (**c** and **d**) polarity gradually becomes symmetrical on upper and lower sides of the plasma membrane 2 h after ‘flipping’ (**d**). Scale bar, 5 μm. *n* = 25–31 roots for each timepoint for **b** and **d** from 3 biologically independent experiments. One-way ANOVA followed by post hoc Tukey’s HSD test revealed *F* stat (3) = 3.8028 with a *P* value of 0.01027 for **b** and 5.8904 with a *P* value of 0.01456 for **d**. White arrowheads in **a** and **c** denote changes in PIN polarity over time. **e**,**f**, Quantification of PIN3 polarity in transgenic lines expressing non-phosphorylatable (S>A) or phosphomimic (S>D) variant of PIN3–YFP. Scale bar, 15 μm. *n* = 21 roots for each line from 3 biologically independent experiments. One-way ANOVA followed by post hoc Tukey’s HSD test revealed *F* stat (2) = 10.692 with a *P* value of 0.0001. **g**,**h**, PIN3:YFP polarity ratios after 30 min in horizontally flipped lateral roots in transgenic PIN3 phosphovariant lines. PIN3:YFP polarized to the upper side 30 min after flipping in WT PIN3 and PIN3 S>D: YFP phosphomimic lines, but not in the PIN3 S>A:YFP phosphodead line. Scale bar, 10 μm. *n* = 21 roots for each line from 3 biologically independent experiments. One-way ANOVA followed by post hoc Tukey’s HSD test revealed *F* stat (2) = 4.541 with a *P* value of 0.0498. **i**,**j**, Quantification of lateral root GSA phenotypes in *rcn1* and *wag1 wag2* mutants. *rcn1* lateral roots have significantly less vertical lateral roots as compared with WT Ws seedlings (**i**). In contrast*, wag1 wag2* seedlings have significantly more vertical lateral roots than WT Col-0 controls (**j**). *n* = 21 roots for each genotype from 3 biologically independent experiments (**i** and **j**). Statistical analysis was performed using two tailed *t*-tests (**i** and **j**).

**Extended Data Fig. 3:**
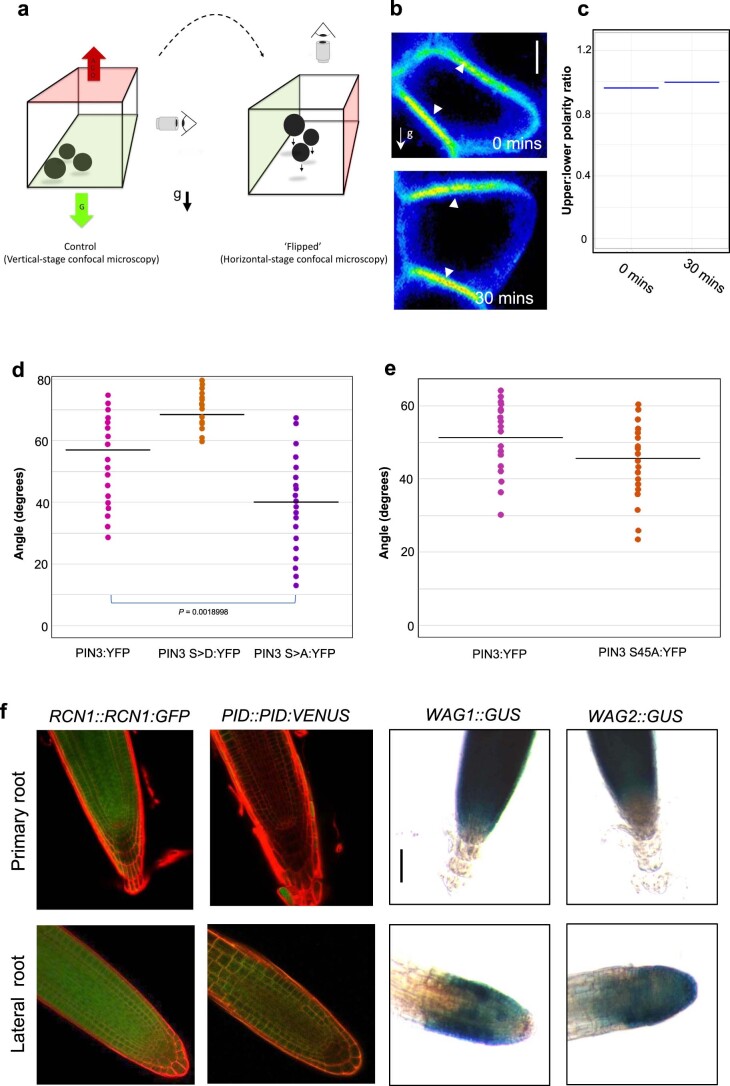
Phosphorylation influences membrane retention kinetics and polarity of PIN proteins in lateral root columella cells. **a**) Schematic representation of the ‘flip’ assay designed to study PIN membrane retention kinetics using vertical stage microscopy. (**b, c**) The PIP1;4:YFP (Wave11_Y) plasma membrane marker remains apolar in columella cells at their GSA (0 mins) or 30 mins after ‘flipping’. Scale bar = 5 μm. White arrowheads denote fluorescent signal at the plasma membrane **(b)**. (**c)** Ratio of PIP1;4:YFP plasma membrane polarity in lateral root columella cells at GSA and 30 mins post ‘flipping’. n = 12–15 roots for each time point from 3 biologically independent experiments. (**d**) Quantification of GSA phenotypes in lateral roots of 12-day-old PIN3:YFP S > A and S > D phosphovariant lines. (**e**) Quantification of lateral root GSA in the 12-day-old seedlings of PIN3:YFP D6PK phosphovariant line (PIN3:S45A:YFP). n = 15–21 roots for each genotype from 3 biologically independent experiments for (**d**) and (**e**). One way ANOVA with an F stat (2) = 7.7295 revealed a p value = 0.0026 for (**d**) while, a two tailed T-test revealed no significant differences were observed as compared to the PIN3:YFP control (**e**). (**f**) Expression of PP2AA/RCN1 phosphatase subunit and the PID/WAG kinase family in primary and lateral roots. *RCN1::RCN1:GFP* is expressed in both, the primary and lateral root columella cells (left upper and lower panels). In contrast *PID::PID:VENUS* and *WAG1::GUS* are not expressed in the primary or lateral root columella (Centre upper and lower panels). *WAG2::GUS* is absent from the primary root columella, but strongly expressed in the lateral root columella. Scale bar = 30 μm. The experiment was repeated independently three times with similar results.

The subcellular distribution of PIN proteins is regulated via cycles of endocytosis and polar or apolar redelivery to the plasma membrane^34^. To understand if there are differences in PIN stabilization within the upper and lower side membranes of lateral root statocytes at GSA, we designed an assay to capture the dynamics of PIN protein relocalization during the gravity-induced repolarization of the cell. This involved ‘flipping’ lateral roots growing at their GSA by 90° within their axis of growth, simply by moving from vertical- to horizontal-stage confocal microscopic imaging and analysing, over time, the faces of the statocyte that were previously ‘up’ and ‘down’ relative to gravity before the ‘flip’ (Extended Data Fig. 3a). For both PIN3:GFP and PIN7:GFP, the ratio of fluorescence signals at the faces of the columella cells that were originally upper and lower with respect to gravity before the experiment were recorded immediately after flipping and then at 30 min intervals for 2 h. Consistent with previous experiments (Fig. 2a–c), we found that, immediately following the flip, PIN3:GFP showed polarity distribution slightly towards the former lower columella cell membrane, (Fig. 3a,b), while the opposite was evident for PIN7:GFP (Fig. 3c,d). Thirty minutes after ‘flipping’ we found that the majority of lateral roots now displayed a significantly higher PIN3 and PIN7 signal at the former upper side of the cell (Fig. 3a–d). This indicates that lower side PINs are endocytosed and polarized in the new direction of gravity and statolith sedimentation at a faster rate as compared with upper side PINs. Comparing the polarity distribution throughout the course of the experiments, we found that, for both PIN3 and PIN7, the proportion of lateral roots with upper polarity gradually decreased, and the majority of lateral roots had acquired a symmetrical distribution across both cell sides 2 h after flipping (Fig. 3a–d). As a control, we performed the same assay with another plasma membrane protein marker line, WAVE_11Y, consisting of the plasma membrane protein PIP1;4 with a C-terminal YFP tag^35^. In lateral roots at their GSA, there was no asymmetry in PIP1;4:YFP expression across the upper and lower membranes of columella cells (Extended Data Fig. 3b,c). Additionally, ‘flipping’ did not lead to the generation of any asymmetry of the YFP signal across the cell, suggesting that the ability to repolarize in the direction of gravity is not a general property of plasma membrane proteins. Taken together, these results indicate that there is differential stability or dynamics of PIN3 and PIN7 at the upper versus lower plasma membranes of lateral root statocytes growing at GSA, with PIN3 and PIN7 being retained at the upper sides for longer.

### PIN phosphorylation affects PIN polarity and redistribution kinetics in lateral root statocytes

The phosphorylation of specific serine (S) residues in the cytoplasmic loops of PIN1 and PIN2 proteins has been shown to induce localization to the shootward (upper) plasma membrane in epidermal and vascular cells, while their dephosphorylation causes PINs to localize to the rootward (lower) plasma membrane^33,36–38^. To explore whether phosphorylation might play a role in the subcellular distribution of PINs within the lateral root columella, we analysed lateral root GSA of plants expressing phosphovariant versions of PIN3:YFP. In these lines, known PID/WAG- or D6PK-targeted serine residues were mutated to either a non-phosphosphorytable (phosphodead) alanine (A)^39,40^ or to the phosphomimic amino acid aspartic acid (D)^39^. Previous studies have shown that phosphorylation at the specific residues of S316, S317 and S321 for PID/WAG and S215 and S283 (annotated as S4 and S5) for D6PKs can affect the gravity-induced repolarization of PIN3 in the root and hypocotyl respectively^39,40^. We found that lines in which the S316, S317 and S321 PID/WAG sites of PIN3:YFP were mutated to alanine (PIN3S>A:YFP) had more vertical lateral root GSAs, while the mutation of those same residues to aspartic acid (PIN3S>D:YFP) induced lateral roots to grow at a more horizontal GSA than control PIN3:YFP plants (Extended Data Fig. 3d). In contrast, the mutation of D6PK phosphosites to alanine (PIN3:S45A:YFP) had no effect on lateral root GSA (Extended Data Fig. 3e).

To investigate the GSA phenotypes of these PIN3 phosphovariant lines we imaged lateral roots growing at GSA and observed that, compared with native PIN3:YFP, the distribution of PIN3S>D:YFP was shifted significantly towards the upper membrane of lateral root columella cells, while that of PIN3S>A:YFP was shifted slightly, but not significantly towards the lower membrane (Fig. 3e,f). We also performed flip assays using the PIN3:YFP phosphovariant derivatives, which showed that the characteristic persistence of a stronger PIN3 signal of the former upper side of lateral root statocyte at 30 min post-flip was lost in the PIN3S>A:YFP line but retained in PIN3S>D:YFP (Fig. 3g,h)

The absence of a GSA phenotype in the D6PK phosphovariant line prompted us to focus on the role of the PID/WAG kinases and PP2AA/RCN1 phosphatases in the regulation of PIN-mediated transport from lateral root statocytes. Analysis of transcriptional and translational marker lines showed that, while PID and WAG1 were below the level of detection in primary and lateral root columella cells, a third member of this family, WAG2, is expressed solely in the lateral root columella (Extended Data Fig. 3f). RCN1 is expressed in both primary and lateral root columella cells (Extended Data Fig. 3f). Interestingly, loss of RCN1 function in the *rcn1* mutant causes lateral roots to grow with a significantly shallower GSA (Fig. 3i), while the double loss-of-function mutant *wag1 wag2* induces a more vertical lateral root GSA (Fig. 3j). These data are therefore compatible with the PIN phosphovariant data and the idea that RCN1-mediated phosphatase activity facilitates the ‘downward’ fluxes of auxin from lateral root statocytes, and that kinases such as WAG2, and possibly others that target the PIN3 cytoplasmic loop, facilitate an opposite, ‘upward’ auxin flux.

### Auxin regulates lateral root GSA through a PIN3-specific phosphorylation module

**Fig. 4:**
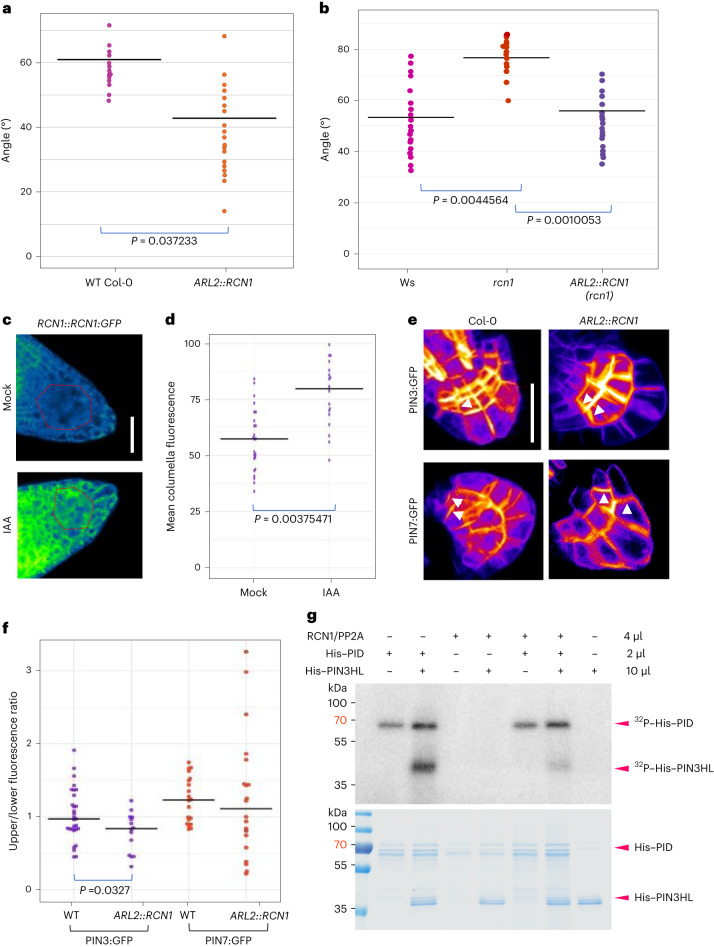
Auxin regulates lateral root GSA through an RCN1-dependent PIN3 module. **a**,**b**, Overexpression of *RCN1* driven by the *ARL2* promoter (ARL2::RCN1) in a WT Col-0 background results in a significantly more vertical lateral root GSA phenotype in contrast to Col-0 control (**a**) and restores the GSA of *rcn1* lateral roots (**b**). *n* = 15–25 for each genotype from 3 biologically independent experiments for both **a** and **b**. Statistical analysis was performed using a two-tailed *t*-test for **a** and a one-way ANOVA with an *F* stat (2) = 19.3276 with *P* = 4.188 × 10^−5^. **c**,**d**, RCN1:GFP protein levels in 10-day-old lateral roots treated with 50 nM IAA for 4 h. Auxin treatment results in a significant increase in GFP signal in the columella cells of RCN1:GFP lateral roots. *n* = 15–21 roots per treatment from 3 biologically independent experiments. Statistical analysis was performed using a two tailed *t*-test (**d**). Red dashed lines represent columella area used for quantification (**c**). Scale bar, 20 μm (**c**). **e**,**f**, Visualisation (**e**) and quantification (**f**) of the effect of overexpression of *RCN1* on PIN polarity in lateral root columella cells. *RCN1* overexpression leads to a significant shift in PIN3:GFP polarity towards the lower side of the cell (**d**). In contrast, PIN7:GFP polarity is unaffected. *n* = 18–24 for each genotype from 3 biologically independent experiments. Statistical analysis was performed using a pairwise two-tailed *t*-test. Scale bar, 15 μm (**e**). **g**, The PP2A/RCN1 subunit is able to dephosphorylate the central hydrophilic loop of PIN3 in vitro. The experiment was repeated independently three times with similar results.

**Extended Data Fig. 4:**
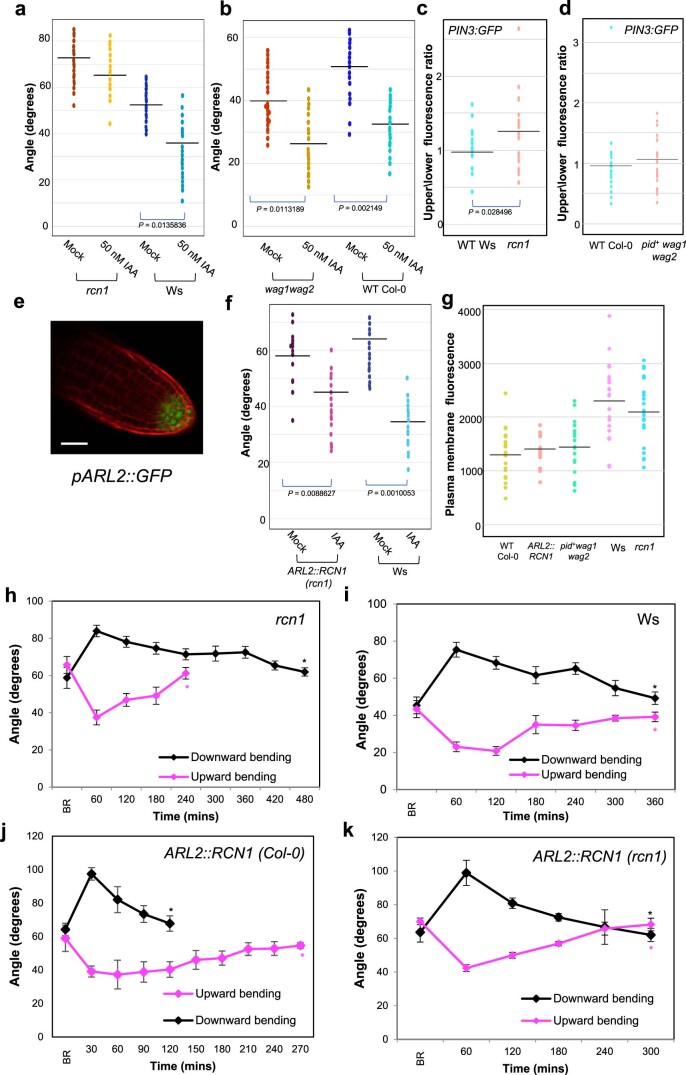
PIN3 dephosphorylation via RCN1 regulates lateral root GSA. (**a,b**) Effect of 50 nM auxin treatment on *rcn1* (**a** and *wag1wag2* (**b**) mutant lateral roots. *rcn1* lateral roots are unaffected by auxin treatment*. wag1wag2* lateral roots adopt a more vertical orientation upon auxin treatment. n = 12–15 roots per genotype per treatment for (A) and (B). One way ANOVA revealed an F stat (3) of 18.0109 and a p value = 5.09X10^−6^ for (**a**) and an F stat (3) = 10.1844 and a p value = 0.0002 for (**b**). (**c, d**) Upper/lower membrane ratios of PIN3:GFP in the columella cells of the *rcn1* and *pid*^*+*^*wag1wag2* mutants. PIN3:GFP polarity is shifted to the upper columella membrane in the *rcn1* background but remains unaffected in the *pid*^*+*^*wag1wag2* background. n = 24–27 roots for each genotype from 3 biologically independent experiments for (**c**) and (**d**). Data was statistically analysed using a two tailed T-test. (**e**) Expression of *ARL2::GFP* in lateral root columella cells. Scale bar = 50 µm. (**f**) Auxin treatment (50 nM IAA) shifts the lateral root GSA of *ARL2::RCN1 rcn1* lateral roots to a significantly steeper orientation. n = 12–15 roots for each genotype per treatment from 3 biologically independent experiments. One way ANOVA revealed an F stat(3) value = 15.8911 with a p value = 3.67X10^−7^. (**g**) Quantification of PIN3:GFP fluorescence in the columella cell plasma membranes in WT and mutant backgrounds. Fluorescence levels did not significantly differ between mutants and their WT controls. n = 22–30 per genotype from 3 biologically independent experiments. (**h**) Stage III lateral roots in the *rcn1* mutant background reorientate upwards to their original GSA in approximately 4 hours, while downward reorientation is delayed. **(i**) Stage III lateral roots of WT Ws plants reorientate both upwards and downwards in 6 hours. (**j**) In contrast, overexpression of *RCN1* in the columella in the WT Col-0 background leads to rapid downward reorientation of stage III lateral roots. (**k**) However, expression of *RCN1* in the *rcn1* mutant background restores reorientation kinetics of stage III lateral roots to a similar pattern as Ws. Data represent average values from 3 independent experiments. Bars represent standard errors of the means (h**-**k) with 6-8 roots reorienting in each direction per experiment per time point.

It has previously been shown that auxin treatment is able to shift lateral root GSA towards a more vertical orientation^16,18,26^. We therefore hypothesized that auxin might affect lateral root GSA by affecting PIN polarity, for example, by increasing the pool of dephosphorylated PINs within the lateral root columella. This increase could be achieved either through an auxin-mediated upregulation of RCN1 expression or activity and/or downregulation of the opposing kinase expression or activity. To explore this possibility, we tested the effect of auxin treatment on lateral root GSA in *rcn1* and *wag1 wag2*. *rcn1* lateral roots failed to respond to auxin treatment (Extended Data Fig. 4a), while the lateral roots of *wag1 wag2* double mutants shifted to a more vertical GSA orientation, similar to WT (Extended Data Fig. 4b), indicating that auxin might control lateral root GSA through an RCN1-dependent pathway. We also observed that PIN3:GFP polarity was shifted towards the upper plasma membrane in the columella cells of the *rcn1* mutant, but remained unchanged from that of WT in the *pid*^*+*^*wag1wag2* mutant background (Extended Data Fig. 4c,d). Consistent with this idea, the overexpression of *RCN1* driven specifically in the columella by the promoter of *ARL2*, a columella-specific gene^41^ (Extended Data Fig. 4e), in the Col-0 background led to a significantly more vertical lateral root GSA phenotype (Fig. 4a). Indeed, this same *ARL2::RCN1* transgene was able to rescue the horizontal GSA phenotype of *rcn1* lateral roots (Fig. 4b) and restore the GSA shift response to auxin treatment in *rcn1* (Extended Data Fig. 4f). Also*, rcn1* stage III lateral roots reoriented upwards towards their GSA more rapidly (Extended Data Fig. 4h,i), while those of *ARL2:RCN1* reoriented downwards more quickly (Extended Data Fig. 4j,k). Importantly, the protein levels of PIN3:GFP did not change significantly from the WT control, in any of these mutant backgrounds (Extended Data Fig. 4g).

**Extended Data Fig. 5:**
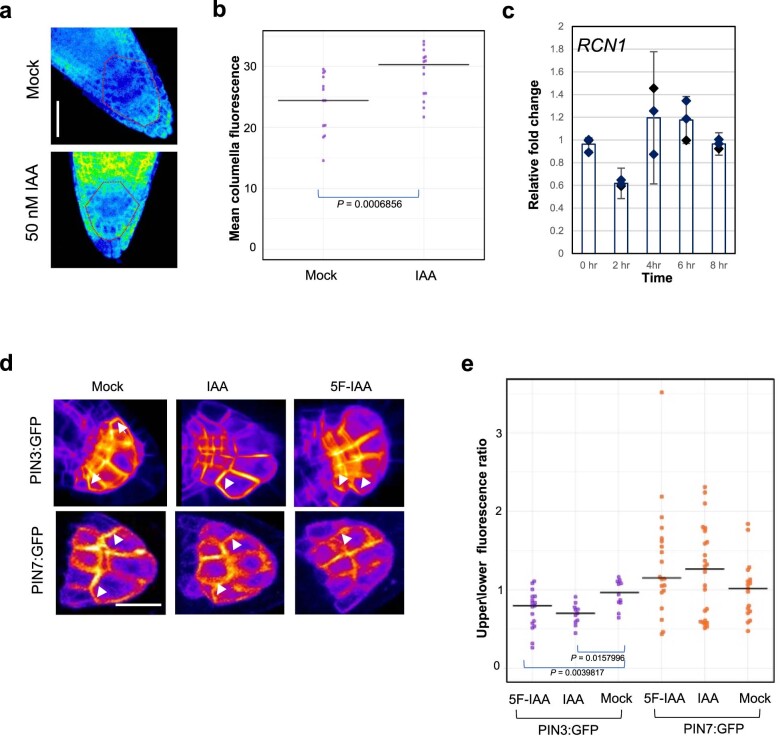
Effect of auxin treatment on *RCN1* expression and protein stability. **a,b**) Effect of auxin treatment for 4 hours on *RCN1::RCN1:GFP* (*PP2AA::PP2AA:GFP* translational reporter line) primary root columella cells. Auxin treatment leads to a significant increase in RCN1:GFP signal levels. n = 15–21 roots from 3 biologically independent experiments. Statistical analysis was performed using a two tailed T-test. Scale bar = 20 µm. Red dashed lines represent area of columella signal quantification in (**a**). (**c**) Effect of 50 nM IAA on *RCN1* transcript levels in lateral root columella cells. No significant increase in *RCN1* levels occurred over an 8 hour time course. Data represent averages from 3 independent experiments with 7-8 root tips harvested for each time point per experiment. Bars represent standard error of the means. (**d,e**) Treatment with 50 nM IAA or 5F-IAA results in a shift in PIN3:GFP polarity towards the lower side of the columella cell, but has no effect on PIN7:GFP polarity. n = 12–15 roots per treatment from 3 biologically independent experiments. One way ANOVA revealed an F stat(5) value = 4.9116 with a P value = 0.0130.

To understand if auxin regulates lateral root GSA directly via RCN1 levels, we analysed the effect of auxin on the abundance of an pRCN1::RCN1:GFP translational reporter by confocal microscopy. We found that treatment with 50 nM Indole Acetic Acid (IAA) for 4 h significantly increased GFP signal in both lateral and primary root columella cells (Fig. 4c,d and Extended Data Fig. 5a,b). Analysis of *RCN1* transcript levels in lateral root tips treated with 50 nM of IAA over a time course between 2 h and 8 h showed that auxin had no effect on *RCN1* expression compared with mock-treated lateral roots (Extended Data Fig. 5c), indicating that the effect of auxin on RCN1 protein levels is post-transcriptional in nature. These data suggest that auxin is able to regulate lateral root GSA through a signalling pathway that is dependent on RCN1 stabilization or enhanced translation.

**Fig. 5:**
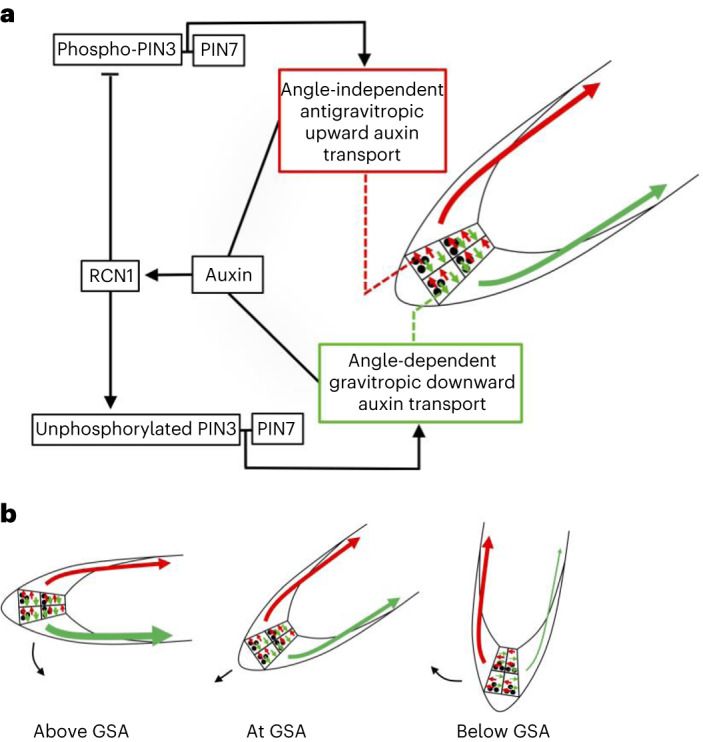
Model of auxin-dependent regulation of GSA. **a**, Model of GSA control in the lateral root in which phosphorylated PIN3 and PIN7 mediate upward, antigravitropic auxin flux from columella cells, while unphosphorylated PIN3 and PIN7 mediate downward, gravitropic auxin transport. In addition to regulating cell elongation further back along the root, auxin also positively regulates levels of the PIN phosphatase subunit RCN1, thereby diminishing the magnitude of AGO. This causes the equilibrium between angle-dependent gravitropic- and angle-independent antigravitropic auxin flux to occur at a more vertical setpoint angle. **b**, Tropic response to displacement either above or below GSA is driven by angle-dependent changes in downward gravitropic auxin flux acting in tension with a more constant, angle-independent upward antigravitropic auxin flux. The thickness of red and green arrows signifies relative auxin flux.

Because the expression of RCN1 solely in the columella had the same effect as exogenous auxin treatment on lateral root GSA, we decided to examine the polarity of PIN3:GFP and PIN7:GFP within the columella cells of lateral roots either in the *ARL2::RCN1* background, or treated with 50 nM of IAA or 5F-IAA for 24 h, an auxin analogue acting specifically through the TIR1 signalling pathway^42^. Under all of these conditions, we found that the polarity of PIN3:GFP was significantly shifted towards the lower side of lateral root columella cells relative to WT or mock controls (Fig. 4e,f and Extended Data Fig. 5d,e). In contrast, the overall polarity of PIN7:GFP was unaffected both in the *ARL2::RCN1* background and by auxin treatment (Figs. 4e,f and 5d,e). These data underline the differences in the subcellular targeting of PIN3 and PIN7 in the gravity-sensing cells. They also indicate that auxin can act to induce more vertical lateral root GSAs by stabilizing RCN1 in the columella, thereby reducing the pool of phosphorylated PIN3 and hence the capacity for upward, antigravitropic auxin flux from the lateral root tip. Consistent with these data, we found that RCN1/PP2A could dephosphorylate the hydrophilic loop of PIN3 in vitro (Fig. 4g). The subsequent reduction in upward auxin flux and the angle dependence of graviresponse in the lateral root means that the equilibrium between gravitropic and antigravitropic auxin fluxes occurs at a smaller angle of displacement from the vertical, producing a steeper GSA.

## Discussion

The ability of plants to maintain their lateral organs at specific GSAs appears to be a complex problem requiring both the monitoring of multiple growth angles and the capacity to reversibly control gravitropic responses both with and against the gravity vector^1,15^. Here we have shown that non-vertical GSAs in lateral roots arise from the interaction of just two phenomena—angle-dependent gravitropic response and an angle-independent antigravitropic offset—mediated at the level of PIN phosphorylation via RCN1, in the gravity-sensing columella cells (Fig. 5a,b).

The demonstration of quantitative, angle-dependent variation in lateral root gravitropic response is significant because while the angle of growth is set by the magnitude of the AGO, it is the capacity of gravitropic response to increase with displacement from the vertical that provides a means to maintain that angle of growth. The central importance of angle dependence in the maintenance of non-vertical GSAs contrasts with its apparent dispensability for achieving vertical growth in primary organs. Although angle dependence contributes to limiting overshooting in primary roots returning to the vertical following displacement^28^, it is perhaps more likely that the major adaptive significance of angle-dependence as a phenomenon lies in its capacity to sustain gravity-dependent non-vertical growth.

Our data have demonstrated that the maintenance of non-vertical GSAs in lateral roots, including both upward and downward growth, is based entirely within a framework of PIN-mediated auxin transport in the lateral root tip (Fig. 1c–f and Extended Data Fig. 1f). Furthermore, because the control of downward gravitropic and upward antigravitropic auxin fluxes from the columella are dependent on the same molecular components, it is the relative magnitude of each that determines angle of growth, independent of the overall levels of auxin and PIN proteins at a given stage of lateral root development.

The concept of gravitropic and antigravitropic activities acting in tension to generate gravity-dependent non-vertical growth becomes less abstract when thought of in the mechanistic terms of the PIN proteins that mediate auxin efflux from the gravity-sensing columella cells. PIN3 and PIN7 in the columella of stage III lateral roots growing at GSA have distinct polarity patterns with PIN3:GFP polarizing slightly to the lower columella cell membrane and PIN7:GFP doing the opposite (Fig. 2a–c). This shifted polarity pattern for PIN7 is reflected in the reorientation kinetics of single and double columella PIN mutants. Lateral roots of *pin3* and *pin3 pin4* mutants display rapid upward bending relative to downward bending, a phenomenon that is lost in the absence of PIN7 (Fig. 2d,f and Extended Data Fig. 2b). While these data indicate that PIN3 and PIN7 make distinct contributions in mediating gravitropic and antigravitropic auxin flux from the columella, they are not exclusive for one or the other. For both PIN3:GFP and PIN7:GFP, reorientation either above and below GSA causes shifts in polarity in lateral root gravity-sensing cells that are consistent with the observed changes in auxin distribution in both downward and upward-bending roots (Extended Data Fig. 2e,f).

During ‘flip assays’ in repolarizing columella cells, both PIN3:GFP and PIN7:GFP were found to persist longer on the former upper side of the statocyte relative to the lower side (Fig. 3a–d). The rapid reduction in PIN3 and PIN7 from the former lower membrane of statocytes in these assays (Fig. 3a–d) demonstrates that the loss of statolith-mediated gravitropic stimulation is associated with a rapid reduction in auxin transport capacity relative to the former upper side of the cell. This is an important finding because it is this response at the cellular level that gives rise to the organ-level response of upward bending where a lateral root is moved back towards the vertical, below its GSA.

In addition to accounting for differences in the kinetics of change in gravitropic and antigravitropic activities, the disparity in PIN protein dynamics on the upper and lower sides of columella cells also suggest a parsimonious model of cellular polarity that avoids the requirement to specify ‘up’ and ‘down’ domains within the cell separately. If statolith sedimentation simply defines a ‘down’ domain in each cell^43,44^, the remainder of the cell can be said to be in a ‘not-down’ state. However, since auxin transport from the lateral plasma membranes of the statocyte is perpendicular to the gravity vector, it is only the relative magnitude of auxin transport from the down and the opposing not-down/up faces of the cells that determine growth trajectory in the vertical plane.

Several molecular and genetic data indicate that the subcellular partitioning of PIN3 to down and not-down regions of columella cells is regulated by the phosphorylation of sites within its cytoplasmic loop, including those targeted by the PID/WAG class of AGC kinases^45^. At this point we cannot distinguish between the effect of the serine to alanine mutations at residues 316, 317 and 321 of PIN3 as being to promote targeting to ‘down’ domains of the cell or to inhibit their phosphorylation-dependent targeting to not-down domains. The same applies in the case of the mutation of these residues to aspartic acid, in that the effect could be either or both, the active targeting of phosphovariant PIN3:GFP to not-down domains or the inhibition of its recruitment to down domains. Whatever the case, these data point to regulatory events involving these phosphosites as being important for PIN protein targeting in the gravity-sensing cells of the root and hence regulation of GSA^39,46^. This conclusion is supported by the finding that the expression of the PIN phosphatase subunit RCN1 in the lateral root columella sufficient to regulate GSA (Fig. 4a,b). In addition to inducing a downward shift in GSA, the overexpression of *RCN1* in the columella also causes a concomitant downward shift in PIN3:GFP but, strikingly, not in PIN7:GFP, again highlighting the differences in the subcellular targeting of these proteins (Fig. 4e,f). At this point the molecular basis of the cell biological differences between PIN3 and PIN7 in lateral root statocytes is not clear. The fact that the subcellular distribution of PIN7 in the columella is unaffected by RCN1 activity suggests that the reason for the distinct upper side polarization of PIN7 is either not related to phosphorylation or, at the very least, involves phosphorylation of sites that are not subject to regulation by RCN1. For PIN3, although phosphorylation of serines 316, 317 and 321 is functionally relevant to the control of its polarity in the columella, we do not know if other phosphosites in its cytoplasmic loop, which are targeted by RCN1, contribute to GSA control.

Previous work has shown that auxin treatment induces steeper GSAs in lateral roots^16,18^. Further, auxin levels in the lateral root tip increase as the lateral root grows out form the main axis, providing an explanation for the increasingly vertical growth of older lateral and a means for the integration of environmental signals controlling root growth angle^26^. Our data show that RCN1 activity in the columella is part of the mechanism underlying this response to auxin. *rcn1* lateral roots are almost entirely resistant to the effect of auxin on GSA (Extended Data Fig. 4a) and auxin treatment increases RCN1 protein levels in the columella (Fig. 4c,d and Extended Data Fig. 5a,b), inducing a downward shift in PIN3:GFP, but not PIN7:GFP (Fig. 4e,f), consistent with the effects of *RCN1* overexpression. Together, these data provide compelling support for the idea that control of RCN1 levels and hence PIN3 polarity in the columella is central to auxin’s ability to regulate lateral root GSA.

## Conclusion

Our work has shown how the patterns of growth angle control observed in lateral roots arise from the interaction of angle-dependent gravitropic response and angle-independent, auxin-repressible antigravitropic offset, both mediated at the level of lateral root columella PIN proteins. This represents a leap in our understanding of the mechanisms governing AGO and of how non-vertical growth can be maintained in plant lateral organs.

Previously, other mechanisms contributing to growth angle control in the young lateral root have been proposed^47^. Here an asymmetry in cytokinin distribution towards the upper side of stage II lateral roots reflecting the lower-side asymmetry in auxin distribution observed during this early, post-emergence phase of growth^47^. In this model, cytokinin inhibits the ability of young lateral roots to respond to gravity, thereby limiting downward growth. This cytokinin-based mechanism pertains to the phase of growth that precedes the capacity of roots to maintain GSAs^15,47^. and so would be expected to influence the growth angle of the lateral root during the first ~0.2–0.5 mm of its growth^15,16,18,47^.

Recent work has also emphasized that other signalling systems are very relevant to the regulation of growth angle. Ogura et al.^22^ used GWAS to identify a role for the EXO70A3 complex in regulating root angle and root system depth through dynamic modulation of the PIN4 auxin efflux transporter. This study is of particular interest because *PIN4* expression across accessions is correlated with rainfall patterns and drought resistance, providing an evolutionary and adaptive link between root architecture control and the environmental conditions. Other interesting studies have further elucidated the role of the LAZY protein family in primary and lateral root gravitropism and growth angle regulation^48–52^. For example, using molecular genetics and structural biology, Furutani et al. have convincingly demonstrated that LAZY proteins interact with RLD proteins, a novel family of regulators of PIN polarity, which in turn leads to the accumulation of PIN3 in lateral root columella cells^52^. These new studies are important and have clearly identified new players in the regulation of lateral root growth angle. The work presented here complements these studies by providing an explanation for the defining property of GSA control, that is, the capacity to maintain an angle relative to gravity by means of both upward and downward tropic response. In this respect, it will be interesting to understand how, for example, LAZY and RLD protein activity integrates with the columella PIN-specific phosphoregulation mechanism identified here. The partial preference for PIN3 to localize to the lower face of columella cells (Fig. 2e) would be consistent with the fact that *lazy* and *rld* mutant lateral roots display more horizontal GSA phenotypes, but there is significant scope for deeper mechanistic links to be uncovered.

Perhaps the most conspicuous open question at hand is a very old one, that of how statolith sedimentation is turned into asymmetry in PIN localization and activity within statocytes. In the context of GSA control, the question relates to understanding the basis of angle-dependent graviresponse, which is so crucial to the maintenance of non-vertical GSAs. By highlighting the evolutionary and adaptive significance of such phenomena, the model of GSA control proposed here provides not only practical tools but also fresh approaches to tackling these fascinating and important questions in plant biology.

## Methods

### Plant materials

All *Arabidopsis* seed stocks are in the Col-0 background unless otherwise stated. R2D2 (ref. ^25^) is in the Utrecht background. The *pin3-3*, *pin7-2*, *pin3-5 pin7-1 [pin3 pin7]*, *pin3-5 pin4-3 [pin3 pin4]*, *pin3-5 pin4-3 pin7-1 [pin3 pin4 pin7]*, *pin4-3 pin7-1 [pin4 pin7]* (refs. ^7,32^), PIN3:GFP, PIN7:GFP^10^*, rcn1* (ref. ^53^), *wag1 wag2* (ref. ^54^), PIN3:YFP, PIN3S>A:YFP and PIN3S>D:YFP^39^, *RCN1::RCN1:GFP [PP2AA::PP2AA:GFP]*, *PID::PID:Venus*, *WAG1::GUS*, *WAG2::GUS*^36^, PIN3:S4S5A:YFP [and PIN3:YFP control]^40^, DII-Venu^24^, *TIR1::TIR1:Venus*, *AFB2::AFB2:Venus* and *AFB3::AFB3:Venus*^26^ lines have been described previously. Ws-0 seeds were obtained from the Nottingham Arabidopsis Stock Centre. The *ARL2:RCN1* construct was generated by cloning 2.5 kb of the *ARL2* promoter upstream of the *RCN1* coding sequence using a multiplex gateway cloning strategy (Invitrogen) into a pALLIGATOR V destination vector. The *ARL2::GFP* construct was generated by cloning 2.5 kB of the *ARL2* promoter sequence into a modified pGreen 0229 vector containing GFP cloned upstream of an NOS terminator. Transformation of these constructs to *Arabidopsis* was accomplished via *Agrobacterium tumefaciens* (strain GV3101)-mediated infiltration by floral dip. The *pid*^*+*^ *wag1 wag2* PIN3:GFP line has previously been described^11^. PIN3:GFP in the *rcn1* and Ws backgrounds and *ARL2::RCN1* PIN3:GFP and *ARL2:RCN1* PIN7:GFP lines were generated by crossing.

### Reorientation experiments

Twelve-day-old seedlings grown vertically on 120 mm square *Arabidopsis thaliana* salts (ATS) medium plates under light and temperature regimes described above were reorientated by appropriate angles in darkness. Images were captured automatically at described intervals using a Canon 700D digital camera and infra-red illumination using the ‘Image Capture’ software in OS El Capitan on a 2013 MacBook Pro to nullify any phototropic effects. The angles of stage III lateral root tips were measured using ImageJ (https://imagej.nih.gov) before reorientation and subsequently at defined time intervals. The average GSAs of reoriented root tips was plotted to generate the reorientation plots. A total of 10–12 roots were used to quantify average GSA at each timepoint for both upward and downward reorientations in each experiment, and each experiment was repeated three times.

### Maintenance of constant gravistimulation and measurement of root orientation

Roots were illuminated with an infra-red light-emitting diode (Radio Shack) and imaged with a charge-coupled device camera interfaced to a computer via a frame grabber card (Imagenation Corp.). A computer feedback system connected to a rotary stage^28^ was used to measure the orientation of the root apex and constrain it to that initial orientation before gravistimulation by making corrections every 45 s. Following reorientation, the root tip was constrained at the new orientation to maintain a constant gravistimulus throughout the experiment. Gravitropic curvature was measured as the rotation of the stage necessary to maintain the root tip at a constant orientation.

### qRT–PCR for *RCN1* expression

RNA was extracted from the lateral and primary root tips of 12-day-old WT Col-0 plants grown on ATS medium overlaid with Sefar Nitex mesh using the Qiagen RNAeasy kit according to the manufacturer’s instructions. Complementary DNA was synthesized from the isolated RNA using oligo dT primers and Superscript II reverse transcriptase (Invitrogen). qPCR was performed using the Bio-Rad CFX Connect Real-Time System (Bio-Rad). GAPDH was used as an internal control.

### Analysis of lateral root GSA

In our experimental conditions, stage III lateral roots are 0.5–3 mm in length and remain at this stage for approximately 24 h. Briefly, the angle that a 1 mm segment of a stage III lateral root made with the vertical was quantified. GSA was plotted as the angle of this segment. At least six roots were quantified for each experiment. Each experiment was repeated three times.

### Confocal microscopy

Ten- to 12-day-old marker seedlings grown on ATS or half strength Murashige and Skoog (MS) medium in standard tissue culture conditions (20–22 °C 16 h day, 8 h dark) were imaged at 20× resolution with the 480 nm and 540 nm lasers using a Zeiss LSM 710 inverted confocal microscope. For vertical stage confocal microscopy and gravistimulation, the imaging setup described in Von Wangenheim et al.^29^ was used. All laser power and gain settings were consistent across images. Briefly, PIN:GFP markers were imaged using a series of *Z* stacks and fluorescence intensity across external membranes was quantified using ImageJ as described in Grones et al.^39^. For flip assays, root tips were counterstained with propidium iodide before imaging. A series of stacks across the central columella was captured for both the GFP and PI channels. Using the ‘Plot profile’ function in ImageJ, the *x*-axis point of maximal intensity in the PI channel was identified as the cell wall. The GFP fluorescence was measured and calculated across each cell membrane on either side of the cell wall. Each experiment was performed three times with at least six roots for each experiment. Representative images were also taken for individual cells across the series of timepoints. The images shown are generated using the ‘Sum of stacks’ function with the ‘16 colours’ LUT. For cell length quantification, WT Col-0 plants on ATS grown on ATS medium were reorientated for a period of 6 h. The entire root system was mounted on a glass slide and counterstained with propidium iodide before imaging the EZ of stage III lateral roots. The length of three to four fully elongated cells epidermal atrichoblast cells on either flank of the root was quantified using ImageJ. The experiment was performed three times with at least six roots at each orientation per experiment. For DII-Venus and R2D2, excluding the lateral root cap, nuclear fluorescence was measured in ten consecutive epidermal cells within the two outermost flanking cell files, beginning from the root tip for each root. Experiments were performed three times with at least ten root tips for each orientation per experiment. For R2D2, nuclear fluorescence intensity was measured across both GFP and mTOMATO channels. For each nucleus, the ratio of GFP/mTOMATO signal was determined. Geometric means and standard errors of the ratios were calculated for both young and older lateral roots. Student’s *t*-test was performed to evaluate statistical differences between the geometric means of the data obtained.

### Recombinant protein expression and purification from *Escherichia coli*

Coding sequences of RCN1, PID and PIN3HL were cloned into the pET28a vector (Novagen) by the restriction enzyme digestion method. Recombinant His-tagged proteins, including His–RCN1, His–PID and His–PIN3HL, were expressed in a BL21 (DE3) strain with induction by 0.5 mM isopropyl β-d-1-thiogalactopyranoside for 16 h at 12 °C. A total of 500 ml of *E. coli* culture was collected by centrifuge, resuspended in 35 ml 1× TBS (50 mM Tris–Cl and 150 mM NaCl; pH 7.6) buffer, and was then subjected to sonication. Proteins were then purified using Ni-NTA His binding resin (Thermo Scientific) following the manufacturer’s instruction. Eventually, the resultant protein samples were checked by sodium dodecyl sulfate–polyacrylamide gel electrophoresis (SDS–PAGE) and visualized by Coomassie brilliant blue staining.

### Isolation of the PP2A/RCN1 complex from plant extracts

A total of 35 ml lysate of His–RCN1 from 500 ml of *E. coli* culture was incubated with 1.5 ml Ni-NTA His binding resin (Thermo Scientific) for 30 min, and the supernatant was discarded. At the same time, 3 g Col-0 seedlings were homogenized into plant extraction buffer (20 mM Tris–HCl, pH 7.5, 150 mM NaCl, 0.5% Tween-20, 1 mM ethylenediaminetetraacetic acid and 1 mM dithiothreitol) containing a protease inhibitor cocktail (cOmplete, Roche) and a protein phosphatase inhibitor tablet (PhosSTOP, Roche). The protein-bound resin was then incubated with protein extracts for 2 h at 4 °C. Afterwards, the column was washed twice with 10 ml wash buffer (1× TBS + 20 imidazole), and then eluted with 1 ml elution buffer (1× TBS + 250 imidazole) three times. Protein samples were checked by SDS–PAGE and visualized by Coomassie brilliant blue staining.

### In vitro (de)phosphorylation assay with γ-[^32^P]-ATP

In vitro (de)phosphorylation assays with γ-[^32^P]-ATP were performed as previously described with some modifications. Recombinant His–PID (1 µl), His–PIN3HL(10 µl) with different concentrations of PP2A were incubated in the reaction buffer (50 mM Tris–HCl pH 7.5, 10 mM MgCl_2_, 1 mM ATP (adenosine 5′-triphosphate) and 1 mM dithiothreitol) at the presence of 5 μCi [γ-^32^P]-ATP (NEG502A001MC, Perkin-Elmer) at 25 °C for 90 min. Afterwards, reactions were stopped by adding the SDS loading dye. The resultant samples were subjected to SDS–PAGE. Gels were developed with a phosphor plate overnight, and the signal was eventually imaged with a Fujifilm FLA 3000 plus DAGE system.

### Statistics and reproducibility

All experiments were repeated independently three times. All statistical data were tested for normality using Kolmogorov–Smirnoff’s test and analysed using either a pairwise two tailed *t*-test or one-way analysis of variance (ANOVA) followed by Tukey’s honestly significant difference (HSD) post hoc tests. Obtained *P* values are presented in each figure, and values for ‘*F*’ along with degrees of freedom, and *P* values from ANOVA tests are described in the appropriate figure legends. Data are presented as individual data points using ‘R’ (Table 1).

**Table 1:** List of primer sequences used in this study

Name	Purpose	Sequence (5′ to 3′)
B5r *RCN1*	*RCN1* entry clone generation	GGG GAC AAC TTT GTA TAC AAA AGT TGT AAT GGC TAT GGT AGA TGA ACC G
*RCN1* B2	*RCN1* entry clone generation	GGG GAC CAC TTT GTA CAA GAA AGC TGG GTT TCA GGA TTG TGC TGC TGT GG
B5r *WAG2*	*WAG2* entry clone generation	GGG GAC AAC TTT GTA TAC AAA AGT TGT AAT GGA ACA AGA AGA TTT CTA TTT CCC TGA C
*WAG2* B2	*WAG2* entry clone generation	GGG GAC CAC TTT GTA CAA GAA AGC TGG GTT TTA AAC GCG TTT GCG ACT CGC
B1 *ARL2*	*ARL2* entry clone generation	GGG GAC AAG TTT GTA CAA AAA AGC AGG CTT TTT AAA CTG ATT ACA AAA ATC TTA TAT AC
*ARL2* B5	*ARL2* entry clone generation	GGG GAC AAC TTT TGT ATA CAA AGT TGT TGT TCA ATA ACA GGT TTT TGT TTC CCA GTT TG
qRCN1 Fwd	*RCN1* qPCR Fwd primer	AGT GTT TGG TGG ACC TGA GC
qRCN1 Rev	*RCN1* qPCR Rev primer	GAT TGT GCT GCT GTG GAA CC

### Reporting summary

Further information on research design is available in the Nature Portfolio Reporting Summary linked to this article.

## Supplementary information

Reporting Summary

## Data Availability

Figures 1–4 in the main manuscript have associated raw data in the form of multiple images used for analysis and generation of graphs. There is no restriction on data availability. All data generated in this study are included within the main text and Supplementary Information. All experimental materials generated in this work are available from the corresponding author upon request. Open access datasets are available at 10.5281/zenodo.8019901 (ref. ^55^). Source data are provided with this paper.

## Source data

Source Data Fig. 1 Unprocessed gel shown in Fig. 4g and unprocessed loading control gel shown in Fig. 4g.
