# Supplementary material for: Antigravitropic PIN polarization maintains non-vertical growth in lateral roots
Source: Nat Plants. 2023 Sep 4;9(9):1500–13. doi: 10.1038/s41477-023-01478-x (PMC10505559; doi:10.1038/s41477-023-01478-x)
Supplement: Supplementary file 1 — Reporting Summary [file 41477_2023_1478_MOESM1_ESM.pdf]

nature portfolio

Corresponding author(s): Prof. Stefan Kepinski

Last updated by author(s): Dec 23, 2022

Reporting Summary

Nature Portfolio wishes to improve the reproducibility of the work that we publish. This form provides structure for consistency and transparency in reporting. For further information on Nature Portfolio policies, see our Editorial Policies and the Editorial Policy Checklist.

Statistics

For all statistical analyses, confirm that the following items are present in the figure legend, table legend, main text, or Methods section.

n/a	Confirmed	
☐	☒	The exact sample size (n) for each experimental group/condition, given as a discrete number and unit of measurement
☐	☒	A statement on whether measurements were taken from distinct samples or whether the same sample was measured repeatedly
☐	☒	The statistical test(s) used AND whether they are one- or two-sided<br>*Only common tests should be described solely by name; describe more complex techniques in the Methods section.*
☒	☐	A description of all covariates tested
☐	☒	A description of any assumptions or corrections, such as tests of normality and adjustment for multiple comparisons
☐	☒	A full description of the statistical parameters including central tendency (e.g. means) or other basic estimates (e.g. regression coefficient) AND variation (e.g. standard deviation) or associated estimates of uncertainty (e.g. confidence intervals)
☐	☒	For null hypothesis testing, the test statistic (e.g. F, t, r) with confidence intervals, effect sizes, degrees of freedom and P value noted<br>*Give P values as exact values whenever suitable.*
☒	☐	For Bayesian analysis, information on the choice of priors and Markov chain Monte Carlo settings
☒	☐	For hierarchical and complex designs, identification of the appropriate level for tests and full reporting of outcomes
☒	☐	Estimates of effect sizes (e.g. Cohen's d, Pearson's r), indicating how they were calculated

Our web collection on statistics for biologists contains articles on many of the points above.

Software and code

Policy information about availability of computer code

Data collection	Data was acquired using the Zeiss LSM Zen Black image acquisition software.
Data analysis	Data was analysed using M/S Excel. Data was analysed using the open source software ImageJ version 2. 9.0 (https://imagej.nih.gov/ij/)

For manuscripts utilizing custom algorithms or software that are central to the research but not yet described in published literature, software must be made available to editors and reviewers. We strongly encourage code deposition in a community repository (e.g. GitHub). See the Nature Portfolio guidelines for submitting code & software for further information.

Data

Policy information about availability of data

All manuscripts must include a data availability statement. This statement should provide the following information, where applicable:

- Accession codes, unique identifiers, or web links for publicly available datasets
- A description of any restrictions on data availability
- For clinical datasets or third party data, please ensure that the statement adheres to our policy

Figures 1-4 in the main manuscript have associated raw data in the form of multiple images used for analysis and generation of graphs. There is no restriction on data availability. All data generated in this study are included within the main text and supplementary information. All experimental materials generated in this work are available from the corresponding author upon request.
Open access datasets are available at 10.5281/zenodo.8019901. A reference to the dataset has been added to the reference list in the main manuscript text file.

Human research participants

Policy information about studies involving human research participants and Sex and Gender in Research.

Reporting on sex and gender	N/A
Population characteristics	N/A
Recruitment	N/A
Ethics oversight	N/A N/A

Note that full information on the approval of the study protocol must also be provided in the manuscript.

Field-specific reporting

Please select the one below that is the best fit for your research. If you are not sure, read the appropriate sections before making your selection.

☒ Life sciences ☐ Behavioural & social sciences ☐ Ecological, evolutionary & environmental sciences

For a reference copy of the document with all sections, see nature.com/documents/nr-reporting-summary-flat.pdf

Life sciences study design

All studies must disclose on these points even when the disclosure is negative.

Sample size	Broadly, sample size for each experiment ranged from 6-12 for each replicate. Experiments were replicated thee times independently. For Fig S1A n = 14-22. For all experiments, 3 biologically independent replicates were performed. Sample size is indicated in the figure legends for each figure. For all experiments, no statistical analysis was performed to predetermine sample size. Instead, sample size was chosen based on standards and previous experience in the field.
Data exclusions	No data was excluded in the analysis.
Replication	Each experiment was replicated three times independently. The data presented in graphs represent the analysis from all three independent experiments.
Randomization	Imaging of samples was randomly performed between control/mock and experimental treatments. Both control and test data were generally acquired at random, except for imaging experiments where a time component was involved. Data analysis for PIN polarization was also performed randomly. The quantification of lateral root gravikinetics and GSA was not analysed randomly as we measured the angles made by a specific segment of the root with reference to gravity, which is not influenced by the observer.
Blinding	Blinding was not performed in this study as imaging capturewas highly time-dependent. Image analysis was blinded, i.e the researchers were not aware if the images they analysed belonged to control or test groups. A subset of the root angle data was analysed through double-blinding resulting in the same distributions as the non-blinded analysis.

Reporting for specific materials, systems and methods

We require information from authors about some types of materials, experimental systems and methods used in many studies. Here, indicate whether each material, system or method listed is relevant to your study. If you are not sure if a list item applies to your research, read the appropriate section before selecting a response.

Materials & experimental systems

n/a	Involved in the study	
☒	☐	Antibodies
☒	☐	Eukaryotic cell lines
☒	☐	Palaeontology and archaeology
☒	☐	Animals and other organisms
☒	☐	Clinical data
☒	☐	Dual use research of concern

Methods

n/a	Involved in the study	
☒	☐	ChIP-seq
☒	☐	Flow cytometry
☒	☐	MRI-based neuroimaging
