# Supplementary figures and images for: Antigravitropic PIN polarization maintains non-vertical growth in lateral roots
Source: Nat Plants. 2023 Sep 4;9(9):1500–13. doi: 10.1038/s41477-023-01478-x (PMC10505559; doi:10.1038/s41477-023-01478-x)

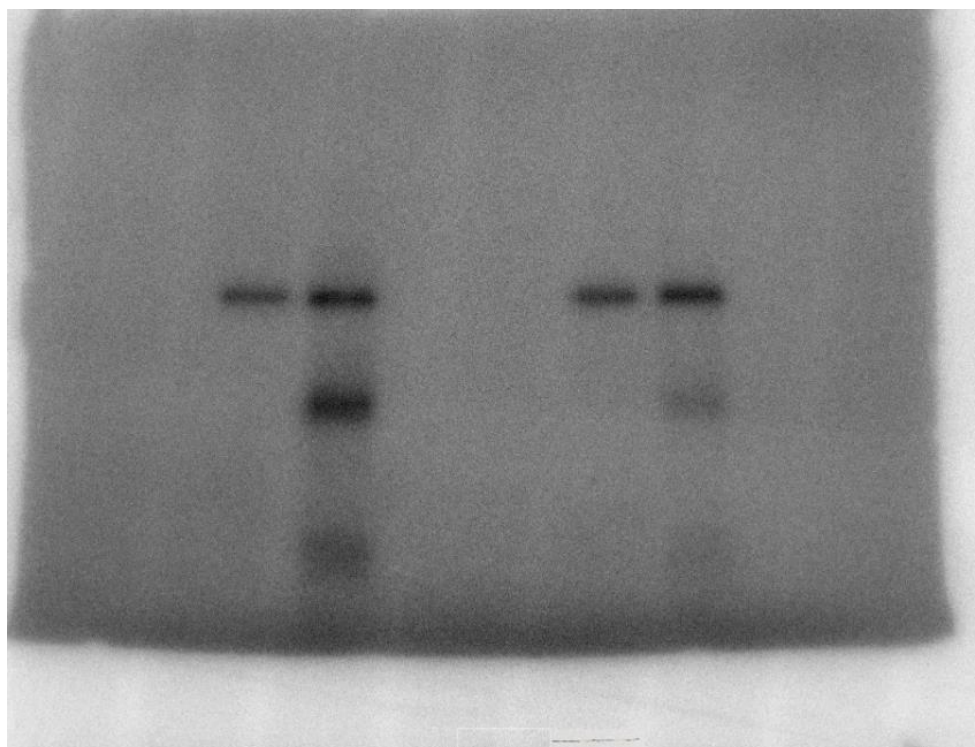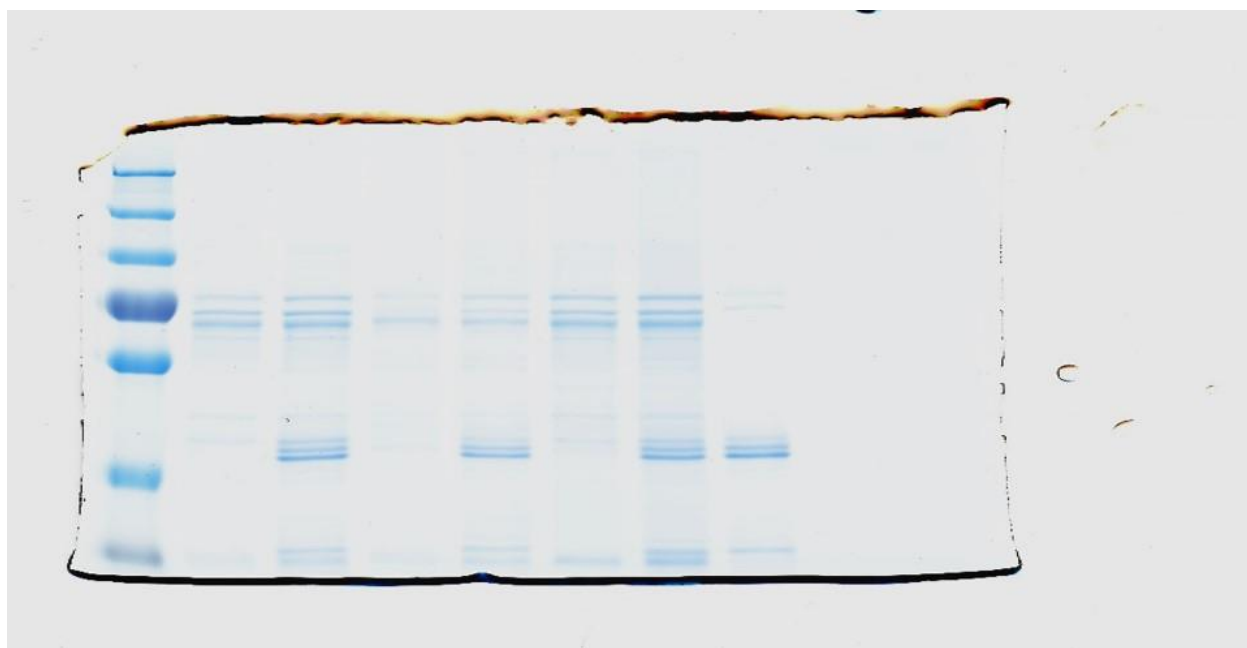

Source gel images for Fig 4G.

Supplement: Source Data Fig. 1 — Unprocessed gel shown in Fig. 4g and unprocessed loading control gel shown in Fig. 4g. [file 41477_2023_1478_MOESM2_ESM.pdf]
